# MicroRNAs in breast cancer—new frontiers in diagnosis, targeted therapy, and prognosis assessment

**DOI:** 10.3389/fonc.2025.1529907

**Published:** 2025-09-01

**Authors:** Jinsuo Xiao, Lanhui Zhang, Ruifan Su, Bo Zhao, Yuanyuan Dang, Chuanlin Zhao, Sujin Wang, Teng Qi, Fuqing Ji

**Affiliations:** ^1^ Northwest University, Xi’an, Shaanxi, China; ^2^ Xi’an NO.3 Hospital, The Affiliated Hospital of Northwest University, Xi’an, Shaanxi, China; ^3^ School of Medicine, Yan’an University, Yan’an, Shaanxi, China; ^4^ School of Medicine, The Chinese University of Hong Kong, Shenzhen, Guangdong, China

**Keywords:** breast cancer, microRNA, diagnosis, targeted therapy, prognosis, biomarkers

## Abstract

Breast cancer is one of the most common malignancies among women globally, with an annually increasing incidence rate. Its complex pathogenesis and high heterogeneity pose significant challenges to clinical diagnosis and treatment. Traditional diagnostic methods and therapeutic approaches have limitations in improving patient survival rates and prognosis, thus urgently necessitating the identification of novel biomarkers and therapeutic targets. MicroRNA (miRNA), a class of endogenous non-coding small RNA molecules with a length of approximately 20–24 nucleotides, finely regulates gene expression by binding to the 3’ untranslated region of target mRNAs, inhibiting gene translation, or promoting mRNA degradation. In 2024, the Nobel Prize in Physiology or Medicine was awarded for research related to miRNA. Numerous studies have demonstrated that miRNAs play pivotal roles in the initiation, progression, invasion, metastasis, and drug resistance of breast cancer. Aberrant expression of specific miRNAs is closely associated with the molecular subtypes, prognosis, and treatment response of breast cancer, suggesting their potential as diagnostic and prognostic biomarkers. To explore the potential value of miRNA in the diagnosis and treatment of breast cancer, this article systematically reviews the latest research progress on the role of miRNAs in the diagnosis and treatment of breast cancer, with a focus on their application as tumor markers in early diagnosis, molecular subtyping, and therapeutic response monitoring. It elucidates the possibilities of miRNAs as therapeutic targets and tools in targeted therapy, including the current research status of miRNA mimics and inhibitors in breast cancer treatment. Furthermore, it analyzes the role of miRNAs in prognosis assessment, exploring their correlation with patient survival rates, recurrence risks, and treatment responsiveness. Additionally, this article discusses the challenges faced by miRNA research in precision medicine for breast cancer and future directions, providing new insights and strategies for early diagnosis, individualized treatment, and prognosis assessment of breast cancer.

## 1 Introduction

Breast cancer is one of the most common malignancies among women worldwide, with an increasing annual incidence rate, posing a severe threat to women’s health (1–3). According to data from the World Health Organization, approximately 2.30 million new cases of breast cancer were reported globally in 2022, ranking second in the global cancer incidence rate (4). The pathogenesis of breast cancer is complex, involving genetic, endocrine, environmental, and other factors, and its high heterogeneity poses significant challenges for diagnosis, treatment, and prognosis (5, 6).

Traditional diagnostic methods for breast cancer, including imaging studies and histopathological analysis, but these methods have certain limitations in early detection and accurate subclassification (7). Additionally, existing treatment strategies such as surgery, radiotherapy, chemotherapy, and targeted therapy, while improving patient survival rates to some extent, still result in recurrence, metastasis, and drug resistance in a considerable proportion of patients (8). Therefore, there is an urgent need to identify new biomarkers and therapeutic targets to improve early diagnosis rates and treatment outcomes for breast cancer.

MicroRNA (miRNA) is a class of endogenous non-coding small RNA molecules with a length of approximately 20–24 nucleotides that regulate gene expression by binding to the 3’ untranslated region of target mRNAs, inhibiting gene translation, or promoting mRNA degradation (9). Since the first discovery of the lin-4 miRNA by Lee et al. in 1993, miRNAs have been proven to play crucial roles in various biological processes, including cell proliferation, differentiation, apoptosis, and metabolism (10), which are critical for multicellular organisms including humans. In the field of cancer research, miRNAs have been found to have dual roles, acting as both tumor suppressors and oncogenes, involved in tumorigenesis and development (11).

In breast cancer, an increasing number of studies have shown that dysregulation of specific miRNAs is closely related to the initiation, progression, invasion, and metastasis of breast cancer (12). For example, miR-21 is highly expressed in breast cancer tissues and promotes tumor cell proliferation and invasion, making it considered an oncogenic miRNA (13). Furthermore, circulating miRNAs, due to their high stability and ease of detection, are considered potential non-invasive biomarkers for early diagnosis and prognosis assessment of breast cancer (14–16).

Given the significant role of miRNAs in breast cancer, this article aims to systematically review the latest research progress on the role of miRNAs in the diagnosis and treatment of breast cancer. Firstly, we will explore the mechanisms of action of miRNAs in the development of breast cancer, including how they regulate tumor cell proliferation, apoptosis, invasion, and metastasis. Secondly, we will discuss the potential of miRNAs as diagnostic biomarkers for breast cancer, with a particular focus on the detection and application of circulating miRNAs. Thirdly, we will assess the prospects for the application of miRNAs in breast cancer treatment, including miRNA-targeted therapeutic strategies and their synergistic effects with traditional therapies. Finally, we will summarize the current research limitations and outlook for the future application of miRNAs in precision medicine for breast cancer. We hope that this review can provide new insights and strategies for early diagnosis, individualized treatment, and prognosis assessment of breast cancer.

## 2 Methods

Articles were searched non-systematically using PubMed, Web of science. We used the following terms: “Breast cancer” and “miRNAs” or “microRNA” or “microRNAs”, as well as “Diagnosis”, “Biomarker” and “Treatment”. The literature search period ranged from 2002 to 2025.

### 2.1 Inclusion criteria

1. Study Type: review articles, clinical trial, article
2. Research Focus: Articles had to primarily focus on the relationship between breast cancer (either denoted as “Breast cancer”) and miRNAs (including “miRNAs”, “microRNA”, or “microRNAs”) in the context of at least one of the following aspects: diagnosis, biomarker discovery, or treatment.

### 2.2 Exclusion criteria

1. Irrelevant Content: Articles that did not directly address the relationship between breast cancer and miRNAs in relation to diagnosis, biomarker, or treatment were excluded.
2. Language and Article Type: Non-English-language articles and any article types other than review articles were excluded.

After an initial screening based on these predefined criteria, a total of 18624 records were identified. Through a meticulous evaluation process, 186 papers that precisely met the research objectives were included in this review. Conversely, 18438 studies were excluded due to their clear irrelevance to the core research questions and aims as defined by the inclusion and exclusion criteria.

## 3 Overview of miRNAs

### 3.1 Biogenesis and functions of miRNA

#### 3.1.1 Biogenesis of miRNAs

**Figure 1:**
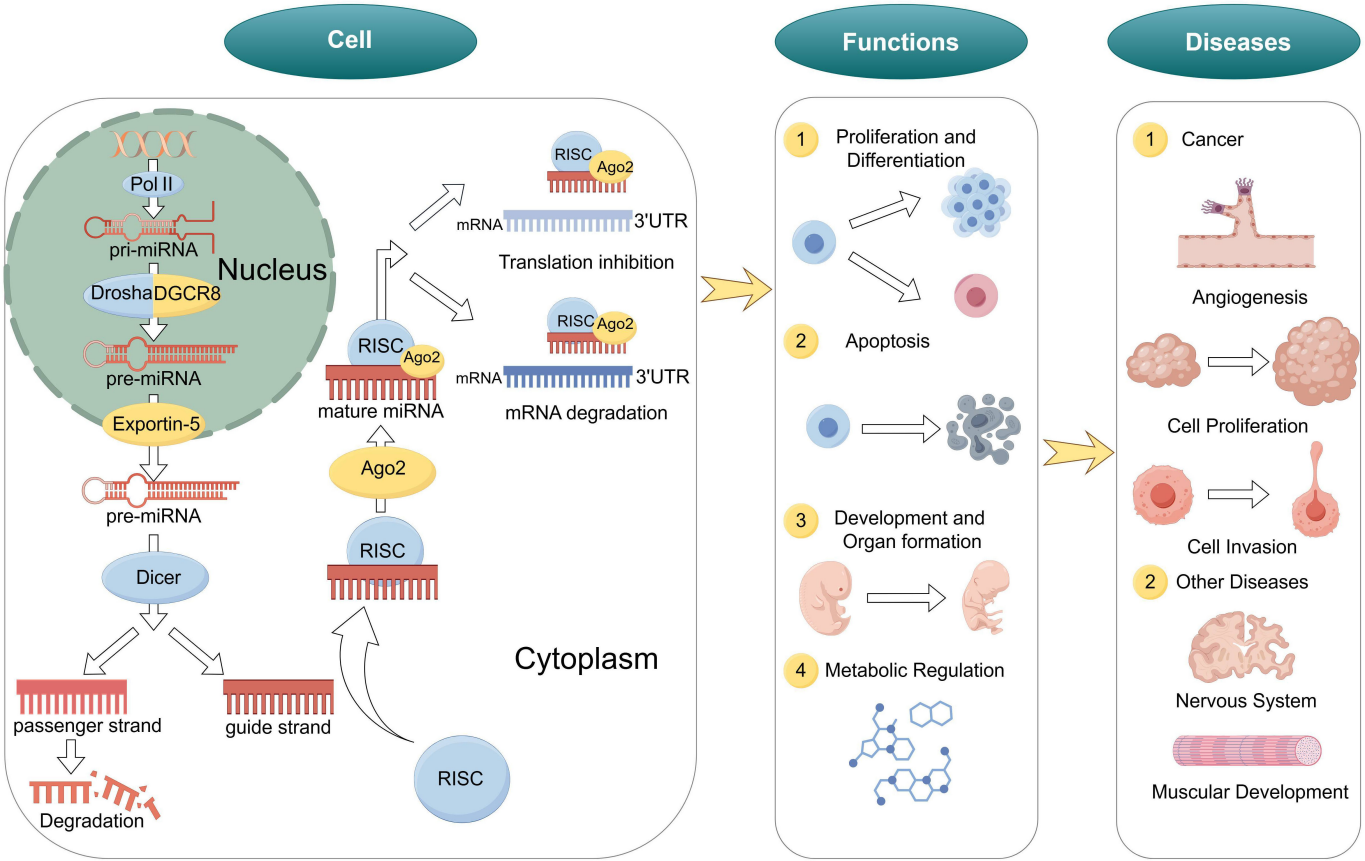
Biogenesis and functions of miRNA. Biogenesis of miRNA, biological functions of miRNAs and role of miRNAs in diseases.

miRNAs are endogenous non-coding single-stranded small RNA molecules, approximately 20–24 nucleotides in length. They silence gene expression at the post-transcriptional level by binding to the 3’ untranslated regions (3’ UTR) of target messenger RNAs (mRNAs) (9, 17). miRNAs are highly conserved in eukaryotes and participate in regulating various biological processes such as cell proliferation, differentiation, apoptosis, and metabolism, playing a crucial role in maintaining normal physiological functions of cells (18, 19) (**Figure 1**).

The biogenesis of miRNAs includes nuclear and cytoplasmic stages (20, 21). Initially, in the nucleus, with hairpin-structured primary miRNAs (pri-miRNAs) are transcribed by RNA polymerase II or III (22). Next, the Microprocessor complex, composed of the RNase III enzyme Drosha and its partner DGCR8, cleaves the pri-miRNA to produce a precursor miRNA (pre-miRNA) of about 70 nucleotides (23).

Subsequently, the pre-miRNA is exported to the cytoplasm via Exportin-5 through the nuclear pore complex (24). In the cytoplasm, the RNase III enzyme Dicer further cleaves the hairpin structure of the pre-miRNA to generate a miRNA duplex of approximately 22 nucleotides (25). One strand of the duplex is selectively loaded into the RNA-induced silencing complex (RISC) to become the mature functional miRNA, while the other strand is degraded (26).

#### 3.1.2 Biological function and mechanism of miRNAs

miRNAs are extensively involved in various biological processes, including:

1. Cell Proliferation and Differentiation: miRNAs regulate cell cycle-related genes, influencing cell proliferation and differentiation (27).
2. Apoptosis: Certain miRNAs can promote or inhibit apoptosis, maintaining tissue homeostasis (28).
3. Development and Organ Formation: miRNAs play key roles in embryonic development and organogenesis, regulating the temporal and spatial specificity of gene expression (29).
4. Metabolic Regulation: miRNAs participate in the regulation of lipid, carbohydrate, and amino acid metabolism, affecting energy balance and the occurrence of metabolic diseases (30).

Mature miRNAs negatively regulate gene expression by binding to the 3’ UTR of target mRNAs (31, 32). Depending on the degree of complementarity between the miRNA and the target mRNA, the regulatory mechanisms can be classified into two types: perfect complementarity leads to mRNA cleavage and degradation, while imperfect complementarity results in translational repression (33). This regulatory mode allows miRNAs to finely control gene expression levels, playing important roles in cell development and function (34).

Additionally, miRNAs can indirectly regulate gene expression through epigenetic mechanisms such as affecting chromatin conformation and DNA methylation (35). Studies have shown that miRNAs interact with other non-coding RNAs, transcription factors, and signaling pathways, forming complex regulatory networks (36).

#### 3.1.3 Role of miRNAs in diseases

Aberrant expression of miRNAs is closely associated with the development and progression of various diseases, especially cancer (37, 38). In tumors, miRNAs can act as oncogenes or tumor suppressors; their dysregulation may lead to uncontrolled cell proliferation, inhibition of apoptosis, promotion of angiogenesis, and metastasis (39). Therefore, miRNAs are considered potential diagnostic biomarkers and therapeutic targets for diseases (40).

### 3.2 The relationship between miRNAs and tumorigenesis and cancer progression

miRNAs have emerged as critical regulators in the complex network of gene expression, significantly influencing tumorigenesis and cancer progression. Aberrant expression of miRNAs can lead to the dysregulation of oncogenes and tumor suppressor genes, thereby contributing to various stages of cancer development, including initiation, proliferation, invasion, metastasis, and resistance to therapy (11, 39).

#### 3.2.1 miRNAs as oncogenes and tumor suppressors

miRNAs can function either as oncogenes (oncomiRs) or tumor suppressors depending on the context of their target genes (41). OncomiRs are typically overexpressed in cancers and promote tumorigenesis by inhibiting tumor suppressor genes. For instance, miR-21 is one of the most studied oncomiRs, frequently overexpressed in breast cancer, and promotes cell proliferation and invasion by targeting PTEN and TPM1 (12, 42). Conversely, tumor-suppressive miRNAs are often downregulated in cancers, leading to the unchecked expression of oncogenes. MiR-34a, a well-known tumor suppressor, is downregulated in various cancers and can induce cell cycle arrest and apoptosis by targeting genes like BCL2 and CDK6 (43, 44).

#### 3.2.2 miRNAs in cell

The balance between cell proliferation and apoptosis is crucial for tissue homeostasis. Dysregulated miRNAs can disrupt this balance, leading to uncontrolled cell growth. Overexpression of miR-155 has been linked to enhanced proliferation in breast cancer cells by targeting the tumor suppressor SOCS1 (45). Additionally, miR-15a and miR-16–1 promote apoptosis by targeting BCL2, and their downregulation has been observed in chronic lymphocytic leukemia (28).

Metastasis is the primary cause of cancer-related deaths. MiRNAs influence metastasis by modulating epithelial-mesenchymal transition (EMT), a process critical for cancer cell invasion and dissemination (46). MiR-10b promotes breast cancer metastasis by targeting HOXD10, leading to increased expression of the pro-metastatic gene RHOC (47).

## 4 Mechanism of action of miRNA function in breast cancer

### 4.1 Expression profile changes of miRNAs in breast cancer

miRNAs play a pivotal role in the regulation of gene expression and have been implicated in the pathogenesis of various cancers, including breast cancer. Alterations in miRNA expression profiles have been observed in breast cancer tissues and cell lines, suggesting their involvement in tumor initiation, progression, and metastasis (48).

Several studies have performed comprehensive miRNA expression profiling to identify miRNAs that are differentially expressed in breast cancer compared to normal breast tissue. Iorio et al. conducted one of the earliest studies, identifying a set of miRNAs consistently deregulated in breast cancer samples (12). They reported overexpression of oncomiRs such as miR-21, miR-155, and downregulation of tumor suppressor miRNAs like miR-125b and miR-145. miR-21 promotes the growth, invasion and metastasis of tumor cells by affecting TGFB, a multifunctional cytokine. miR-125b downregulation may contribute to tumor initiation and progression by affecting target genes such as YES, ETS1, TEL, AKT3 which can lead to impaired differentiation capacity of cancer cells. Blenkiron et al. identified miRNA signatures associated with estrogen receptor (ER), progesterone receptor (PR), and HER2 status (49). For instance, miR-210 was found to be upregulated in HER2-positive tumors. Interestingly, Wu et al. demonstrated that plasma levels of miR-205 and miR-155 were significantly elevated in breast cancer patients compared to healthy controls (50). Correspondingly, treatment modalities can also influence miRNA expression in breast cancer cells. Adams et al. observed that miR-206 expression was upregulated in breast cancer cells treated with anti-estrogen therapies, suggesting a role in endocrine resistance (51).

Epigenetic mechanisms, such as DNA methylation and histone modifications, can regulate miRNA expression in breast cancer. Mulrane et al. performed integrated miRNA and mRNA expression profiling to identify epigenetically deregulated miRNAs in breast cancer cell lines (52). They found that hypermethylation of miRNA gene promoters led to decreased expression of tumor-suppressive miRNAs, contributing to tumor progression. Similarly, Liu et al. found that M6A modification is involved in miRNA biogenesis, and aberrant M6A modification may aggravate tumor progression (53).Pan et al. revealed that methyltransferase-like 3 (METTL3) accelerates the maturation of miR-221-3p and enhances the expression of miR-221-3p by increasing the M6A modification of pri-miR-221-3p, while promote the resistance of breast cancer cells to adriamycin(ADR) through the METTL3/miR-221–3 p/Hipk2/Che -1 axis. Conversely, inhibition of miR-221-3p decreased tumor growth and reversed METTL3 overexpression-induced drug resistance (54).

### 4.2 miRNA regulation of proliferation and apoptosis in breast cancer cells

**Figure 2:**
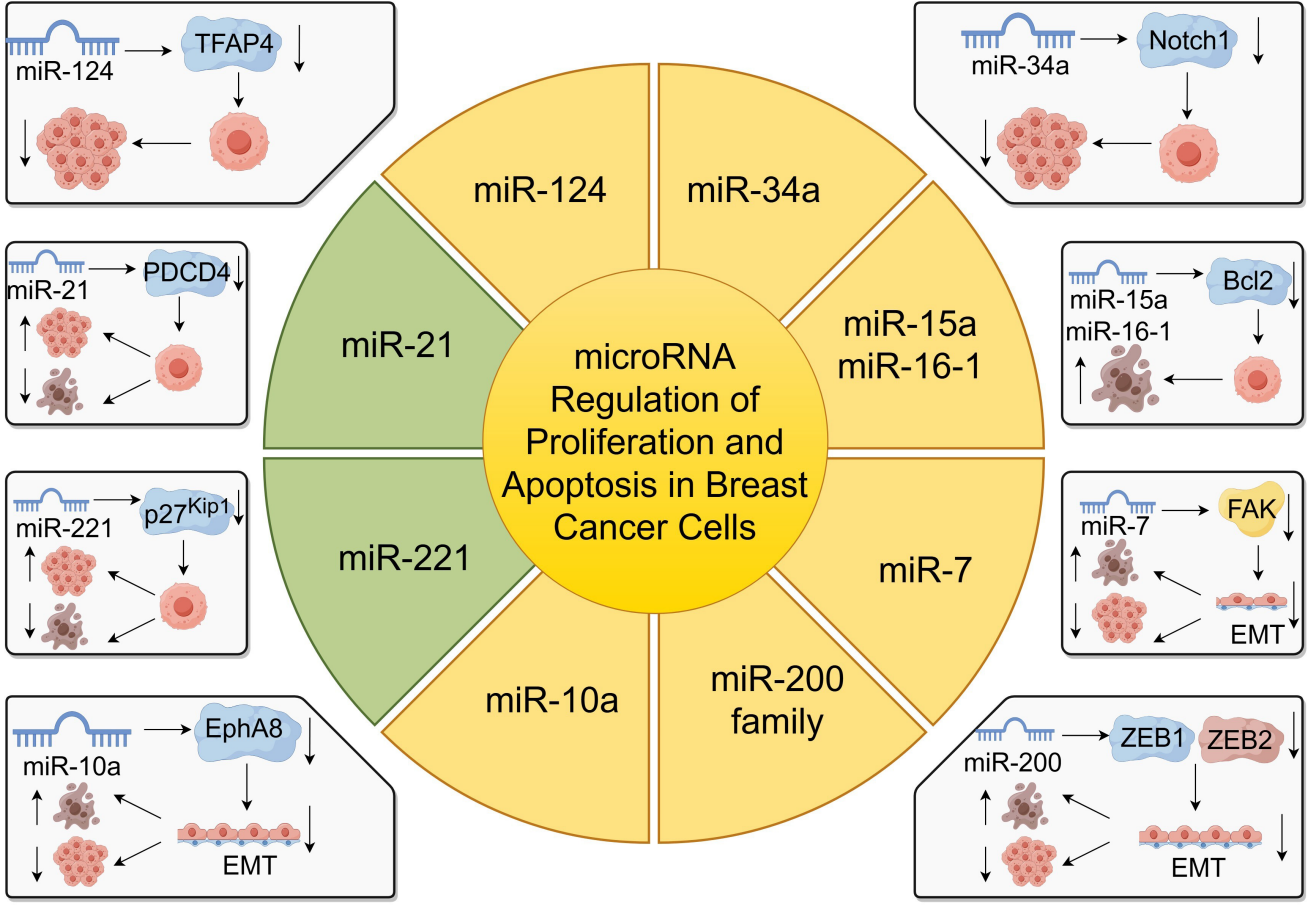
miRNA regulation of proliferation and apoptosis in breast cancer cells.

miRNAs play a pivotal role in regulating gene expression and have been critically implicated in breast cancer progression by modulating cell proliferation and apoptosis (55, 56). By targeting specific mRNAs, miRNAs can function as oncogenes or tumor suppressors, thereby influencing tumor growth and development (**Figure 2**).

For instance, miR-124 has been identified to target the transcription factor activating enhancer-binding protein 4 (TFAP4), leading to the inhibition of growth and invasion of breast cancer cells (57). Overexpression of miR-124 significantly reduces cell proliferation and promotes apoptosis, suggesting its potential as a therapeutic target (57). Similarly, miR-218 suppress cancer progression by targeting the 3’-UTR regions of CDK6 and cyclin D1, as demonstrated in gastric cancer studies, indicating a possible analogous mechanism in breast cancer (58).

miR-34a functions as a tumor suppressor by regulating genes involved in apoptosis, such as Notch1, leading to increased apoptosis in breast cancer cells (59). On the contrary, miR-155 is overexpressed in breast cancer and acts as an oncogenic miRNA by promoting cell proliferation and inhibiting apoptosis (12).

miR-21, another oncogenic miRNA, is commonly overexpressed in breast cancer and facilitates tumor cell proliferation while inhibiting apoptosis by targeting tumor suppressor genes like PDCD4 (60). The miR-200 family also plays a significant role in regulating EMT, thus affecting proliferation and apoptosis in breast cancer cells (61).

Moreover, miR-7 inhibits epithelial-to-mesenchymal transition (EMT) and metastasis of breast cancer cells by targeting focal adhesion kinase (FAK) expression (62). The upregulation of miR-7 suppresses proliferation and induces apoptosis in breast cancer cells (62). Additionally, miR-10a has been shown to regulate migration and invasion in glioma through EMT modulation via EphA8, suggesting its potential involvement in breast cancer metastasis (63).

Furthermore, miR-221 and miR-222 promote proliferation by targeting cell cycle regulators such as p27^Kip1^ and estrogen receptor alpha (ERα), with their upregulation correlating with increased invasiveness and poor prognosis in breast cancer patients (64). Conversely, miR-15a and miR-16–1 induce apoptosis and inhibit tumor growth by targeting the anti-apoptotic gene BCL-2 (28).

### 4.3 miRNA regulation of breast cancer cell invasion and metastasis

**Figure 3:**
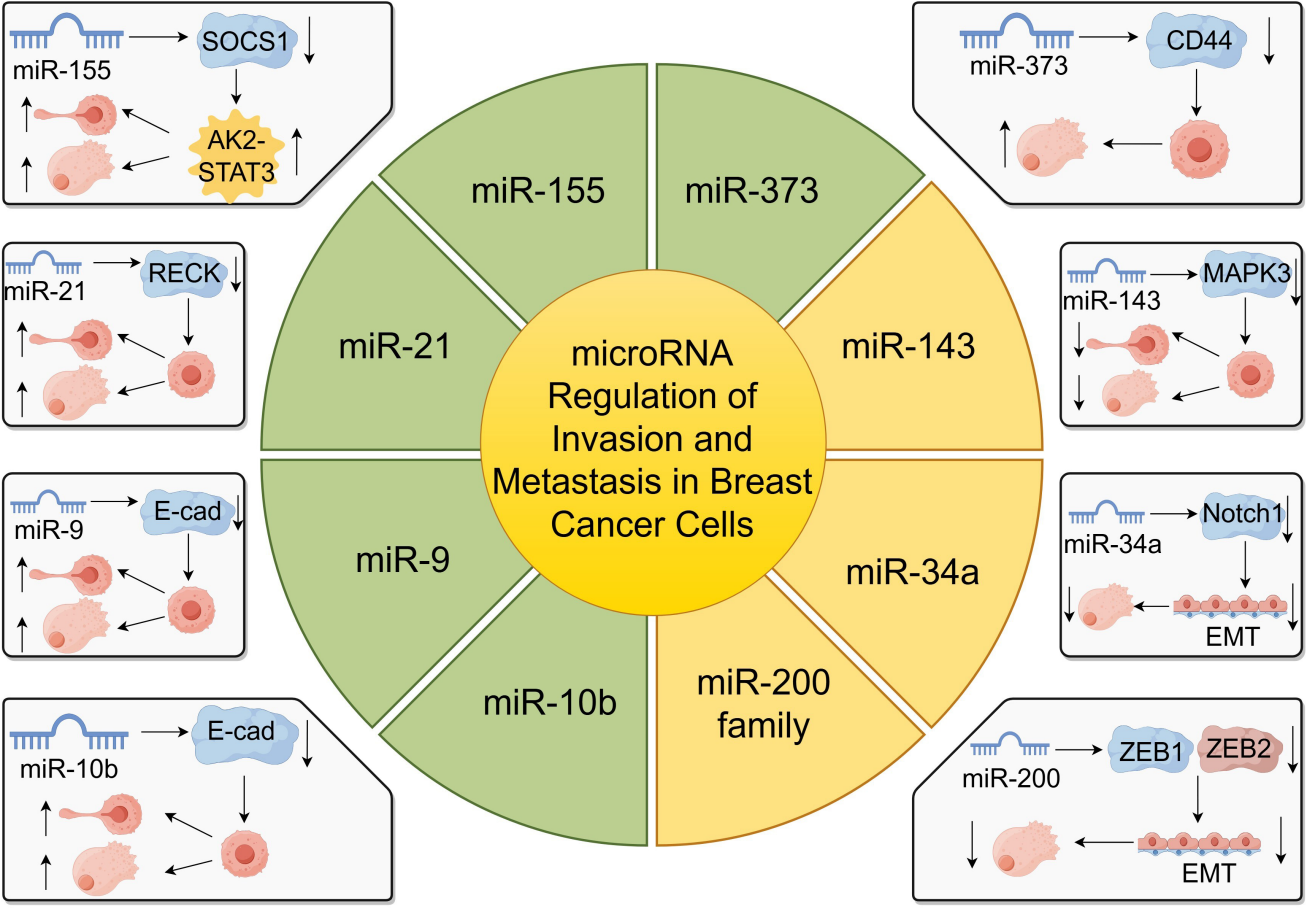
miRNA regulation of breast cancer cell invasion and metastasis.

miRNAs play critical roles in the regulation of gene expression involved in cancer progression, particularly in the invasion and metastasis of breast cancer cells (65, 66). These small non-coding RNAs can function as oncogenes or tumor suppressors by targeting mRNAs that encode proteins essential for cell adhesion, migration, and invasion (**Figure 3**).

One of the most studied miRNAs in breast cancer metastasis is miR-10b. Liu et al. demonstrated that miR-10b targets E-cadherin, a key cell-cell adhesion molecule, thereby promoting breast cancer metastasis (65). The downregulation of E-cadherin leads to decreased cell adhesion and increased cell motility, facilitating metastatic spread (65). Similarly, Ma et al. found that therapeutic silencing of miR-10b in a mouse mammary tumor model significantly inhibited metastasis without affecting primary tumor growth, highlighting miR-10b as a potential therapeutic target (47, 66).

miR-125b has been implicated in breast cancer invasion and chemoresistance. Wang et al. reported that circulating miR-125b serves as a marker for predicting chemoresistance in breast cancer patients (67). Elevated levels of miR-125b are associated with enhanced invasion and poor response to chemotherapy, suggesting its role in metastasis and treatment outcomes (67).

Another tumor-suppressive miRNA, miR-143, is often downregulated in breast cancer tissues. Du et al. showed that miR-143 suppresses tumor proliferation and bone metastasis by targeting MAPK3 (68). Restoration of miR-143 levels led to inhibited cell migration and invasion, underscoring its potential as a therapeutic agent (68).

miR-373 and miR-520c have been identified as promoters of tumor invasion and metastasis. Huang et al. demonstrated that these miRNAs enhance metastatic capabilities by downregulating tumor suppressor genes and activating pro-metastatic genes. Specifically, they target genes involved in cell adhesion and cytoskeletal rearrangement, facilitating tumor cell dissemination (69, 70).

Additionally, miR-21 is overexpressed in breast cancer and contributes to invasion and metastasis by targeting tumor suppressor genes such as PTEN and RECK (71, 72). This overexpression correlates with poor prognosis and increased metastatic potential (71). miR-155 also promotes metastasis by targeting SOCS1, leading to the activation of the JAK2/STAT3 signaling pathway, which enhances cell migration and invasion (45, 73).

Conversely, the miR-200 family functions as metastasis suppressors by inhibiting epithelial-to-mesenchymal transition (EMT), a process crucial for cancer metastasis (61, 74). They target transcriptional repressors ZEB1 and ZEB2, which are key regulators of EMT (74). Loss of miR-200 expression is associated with increased invasiveness and metastatic potential (61).

miR-34a acts as a tumor suppressor by targeting genes involved in EMT and metastasis, such as Notch1 and Fra-1, thereby reducing cell invasion and metastasis (75, 76).

Moreover, miR-9 promotes metastasis by directly targeting E-cadherin and activating β-catenin signaling, which enhances cell motility and invasiveness (77). High miR-9 expression levels correlate with advanced tumor stages and poor patient prognosis (77).

## 5 Application of miRNA in breast cancer diagnosis

### 5.1 miRNAs as diagnostic biomarkers in breast cancer

Early detection of breast cancer significantly improves patient prognosis and survival rate (78). Traditional diagnostic methods, such as mammography and tissue biopsy, have limitations including invasiveness, cost, and sometimes insufficient sensitivity and specificity (79). Therefore, there is a pressing need for non-invasive, reliable biomarkers for early breast cancer detection. miRNAs, small non-coding RNAs involved in post-transcriptional gene regulation, have emerged as promising diagnostic biomarkers due to their stability in bodily fluids and their altered expression profiles in cancer patients (14, 80).

Circulating miRNAs in serum and plasma have been extensively studied as potential diagnostic tools for breast cancer. Shimomura et al. identified a novel combination of serum miRNAs, including miR-1246 and miR-1307-3p, which could effectively detect breast cancer at early stages (81). Their study demonstrated that these miRNAs had high sensitivity and specificity, suggesting their utility in clinical diagnostics (81).

Similarly, Wu et al. employed next-generation sequencing to profile miRNAs in breast cancer patients, identifying several miRNAs with differential expression that could serve as potential biomarkers (50). Among these, miR-21 and miR-221 were significantly upregulated in breast cancer patients compared to healthy controls (50), consistent with previous reports linking these miRNAs to breast cancer development (12, 82).

Eichelser et al. reported deregulated serum concentrations of circulating cell-free miR-17, miR-34a, miR-155, and miR-373 in breast cancer patients (83). Their findings indicated that these miRNAs could reflect tumor dynamics and might be used to monitor disease progression (83). Moreover, miR-155 has been widely studied and is considered a key player in breast cancer pathogenesis (84, 85).

Kodahl et al. identified a novel circulating miRNA signature as a potential non-invasive multi-marker test in estrogen receptor-positive early-stage breast cancer (86). Their case-control study suggested that a combination of miRNAs could improve diagnostic accuracy (86).

In addition to these studies, other miRNAs have been identified as potential diagnostic biomarkers. Heneghan et al. demonstrated that miR-195 was significantly elevated in the blood of breast cancer patients, distinguishing them from healthy individuals (87). Similarly, Chan et al. identified a panel of circulating miRNAs, including miR-145 and miR-451, that could serve as biomarkers for breast cancer detection (88).

The stability of miRNAs in circulating blood is attributed to their encapsulation within exosomes or association with RNA-binding proteins, protecting them from RNase degradation (89). This property enhances their potential as reliable biomarkers (89, 90).

Despite promising results, there are challenges in translating miRNA biomarkers into clinical practice. Variability in miRNA expression due to sample processing, patient heterogeneity, and detection methods can affect reproducibility (91). Thus, the Standardized of protocols and larger-scale validation studies are needed to confirm the clinical utility of miRNAs as diagnostic biomarkers (92).

### 5.2 Detection of circulating MiRNAs in blood

Circulating miRNAs in blood have emerged as promising non-invasive biomarkers for the early detection, prognosis, and monitoring of breast cancer (93, 94). Due to their stability in body fluids and their specific expression profiles associated with various pathological conditions, detecting circulating miRNAs has gained significant attention in cancer research (90, 95).

Several analytical techniques have been developed and optimized for the detection and quantification of circulating miRNAs in blood samples. The most commonly used methods include quantitative real-time PCR (qRT-PCR), microarray analysis, and next-generation sequencing (NGS).

qRT-PCR is widely used due to its high sensitivity, specificity, and quantitative capabilities (91). It involves reverse transcription of miRNAs into cDNA followed by amplification using specific primers. This method requires careful normalization using stable endogenous controls or spiked-in synthetic miRNAs to account for variations in sample processing and RNA extraction efficiency (96, 97).

Microarray platforms enable the simultaneous detection of hundreds of miRNAs, providing a comprehensive expression profile (98). However, microarrays have limitations in sensitivity and dynamic range compared to qRT-PCR and NGS. They are useful for initial screening studies to identify differentially expressed miRNAs in patient samples (87).

Next-Generation Sequencing (NGS) allows for high-throughput sequencing of small RNAs, offering the most comprehensive analysis of the miRNA transcriptome, including the discovery of novel miRNAs (99, 100). Wu et al. utilized NGS to profile miRNAs in breast cancer patients, identifying specific miRNAs with potential diagnostic value (50).

Despite technological advances, detecting circulating miRNAs presents several challenges. The low abundance of miRNAs in circulation requires highly sensitive detection methods (101). Additionally, the presence of hemolysis can release intracellular miRNAs from blood cells, confounding results (102). Standardization of sample collection, processing, and data normalization is crucial for obtaining reliable and reproducible results (92, 103).

In breast cancer, numerous studies have focused on detecting circulating miRNAs as diagnostic and prognostic biomarkers. For example, Shimomura et al. identified a panel of serum miRNAs that could effectively detect early-stage breast cancer using qRT-PCR. Other studies have demonstrated the feasibility of using circulating miRNAs to monitor treatment response and disease progression (104, 105).

Silva et al. examined vesicle-related miRNAs in plasma and their correlation with survival in lung cancer patients, highlighting the potential of exosome-associated miRNAs as biomarkers (106). Similar approaches have been applied in breast cancer to isolate exosomal miRNAs, which may offer increased specificity and stability (107, 108).

The detection of circulating miRNAs in blood represents a promising avenue for the development of non-invasive diagnostic tools in breast cancer. Advances in detection technologies and standardization of methodologies will enhance the reliability of circulating miRNAs as biomarkers, potentially improving early detection and patient outcomes.

## 6 miRNA in the treatment of breast cancer

### 6.1 miRNA-targeted therapeutic strategies

miRNAs have emerged as critical regulators in cancer biology, including breast cancer, by modulating gene expression at the post-transcriptional level (109, 110). The dysregulation of specific miRNAs in breast cancer has provided a rationale for developing miRNA-targeted therapeutic strategies aimed at restoring normal miRNA function or inhibiting oncomiRs (111, 112).

miRNA replacement therapy involves restoring the expression of tumor-suppressive miRNAs that are downregulated in cancer cells (113). Synthetic miRNA mimics are designed to replicate the function of endogenous miRNAs. Bader et al. highlighted the potential of miRNA replacement therapy, emphasizing its ability to target multiple oncogenic pathways simultaneously (109). For instance, the restoration of miR-34a, a well-known tumor suppressor, has shown significant anti-tumor effects in preclinical models of breast cancer (114, 115).

The first miRNA mimic to enter clinical trials was MRX34, a liposomal miR-34a mimic, demonstrating the translational potential of this strategy (116). Although the phase I clinical trial was halted due to immune-related adverse events, it provided valuable insights into the challenges and considerations in miRNA therapeutics (116, 117).

oncomiRs can be inhibited using antisense oligonucleotides (anti-miRs), locked nucleic acids (LNAs), or miRNA sponges (118, 119). These molecules are designed to specifically bind to and inhibit the function of target miRNAs. Adams et al. discussed the aberrant regulation and function of miRNAs in cancer and the therapeutic potential of miRNA inhibition (112).For example, inhibition of miR-21, an oncomiR overexpressed in breast cancer, using antisense oligonucleotides resulted in decreased tumor growth and increased apoptosis in preclinical studies (120). Similarly, targeting miR-155 has shown promise in reducing breast cancer proliferation and metastasis (67).

Effective delivery of miRNA therapeutics remains a significant challenge due to issues related to stability, specificity, and off-target effects (121, 122). Various delivery systems have been explored, including lipid nanoparticles, viral vectors, and extracellular vesicles (123, 124).Van Zandwijk et al. conducted a phase I study using miRNA-loaded minicells for patients with recurrent malignant pleural mesothelioma, demonstrating the feasibility of systemic miRNA delivery (110). Although the study was in mesothelioma patients, the approach provides a framework that could be applied to breast cancer therapy.

Several clinical trials are underway to evaluate the safety and efficacy of miRNA-based therapeutics. Cortez et al. emphasized the role of miRNAs in body fluids as both hormones and biomarkers, underlining their potential in cancer therapy (111). The ongoing research focuses on optimizing delivery methods, improving specificity, and minimizing adverse effects (125, 126).

Despite the promising potential of miRNA-targeted therapies, challenges such as immune activation, off-target effects, and delivery efficiency need to be addressed (127). Bouchie highlighted the entry of the first miRNA mimic into clinical trials as a significant milestone, yet also pointed out the hurdles that remain (113).

Future strategies may involve the use of combinational therapies, personalized medicine approaches, and advanced delivery systems to enhance the efficacy of miRNA therapeutics in breast cancer (128, 129).

### 6.2 Synergistic effects of MiRNAs with chemotherapy and radiotherapy

miRNAs have emerged as critical modulators of gene expression in cancer cells, influencing various cellular processes such as proliferation, apoptosis, and response to therapy (94, 130). In breast cancer, the interplay between miRNAs and conventional therapies like chemotherapy and radiotherapy has gained significant attention, as miRNAs can modulate therapeutic efficacy and resistance mechanisms (131, 132).

miR-451 has been implicated in the resistance of breast cancer cells to the chemotherapeutic drug doxorubicin. Kovalchuk et al. found that overexpression of miR-451 in MCF-7 breast cancer cells led to increased sensitivity to doxorubicin by targeting the multidrug resistance protein MDR1 (133). Conversely, downregulation of miR-451 contributed to chemoresistance, indicating its role in modulating drug response (133).

Wang et al. reported that miR-122 inhibits cell proliferation and tumorigenesis in breast cancer by targeting the insulin-like growth factor 1 receptor (IGF1R) (134). IGF1R is known to confer resistance to chemotherapy and radiotherapy (135). By targeting IGF1R and regulate PI3K/Akt/mTOR/p70S6K pathway,miR-122 plays an important role in inhibiting the tumorigenesis (134).

miR-21 is frequently overexpressed in breast cancer and is associated with poor prognosis and therapy resistance (136, 137). Wang et al. highlighted miR-21 as a novel therapeutic target, as its overexpression promoted breast cancer cell proliferation and metastasis *in vivo* by targeting Leucine zipper transcription factor-like 1 (LZTFL1) (138). miR-21 regulates several targets involved in apoptosis and DNA repair, such as PTEN and PDCD4, influencing the response to therapy (71, 139).

The tumor microenvironment plays a crucial role in therapy resistance (140). Mao et al. discussed how stromal cells and miRNAs within the tumor microenvironment affect breast cancer progression and response to treatment (141). miRNAs can modulate the communication between cancer cells and stromal cells, impacting the efficacy of chemotherapy and radiotherapy (142).

miRNAs can influence therapy response through various mechanisms:

1. Regulation of Apoptosis: miRNAs like miR-34a promote apoptosis in response to DNA damage induced by chemotherapy or radiotherapy (143).
2. Modulation of Drug Transporters: miR-451 affects the expression of drug transporters like MDR1, altering drug accumulation in cancer cells (133).
3. DNA Repair Pathways: miR-155 and miR-21 modulate DNA repair genes, impacting the effectiveness of DNA-damaging agents (137, 144).
4. Epithelial-to-Mesenchymal Transition (EMT): miR-205 inhibits EMT, which is associated with therapy resistance (145).

Understanding the role of miRNAs in therapy response opens avenues for combination treatments. Therapeutic strategies could involve:

1. miRNA Mimics: Introducing tumor-suppressive miRNAs to enhance sensitivity to therapy (146).
2. Anti-miRNAs: Inhibiting oncomiRs like miR-21 to reduce resistance (147).
3. Biomarkers for Predicting Response: Profiling miRNA expression to tailor personalized treatment plans (90).

miRNAs significantly impact the efficacy of chemotherapy and radiotherapy in breast cancer by modulating key pathways involved in drug response and resistance. Targeting specific miRNAs offers a promising strategy to enhance therapeutic outcomes and overcome resistance.

## 7 miRNA role in prognosis assessment of breast cancer

### 7.1 The relationship between miRNA and survival rates in breast cancer patients

Breast cancer remains one of the leading causes of cancer-related mortality among women worldwide (78). Recent studies have highlighted the crucial role of miRNAs in cancer progression and patient prognosis (12, 36). miRNAs are small non-coding RNAs that regulate gene expression post-transcriptionally and have been implicated in various cellular processes, including proliferation, apoptosis, and metastasis (55). This section explores the relationship between specific miRNAs and the survival rates of breast cancer patients.

miR-29a has been shown to promote migration and invasion in breast cancer cells by targeting ten-eleven translocation 1 (TET1), thereby activating epithelial-mesenchymal transition (EMT) (148). Pei et al. reported that elevated levels of miR-29a are associated with poor overall survival in breast cancer patients, suggesting its potential as a prognostic biomarker (148).

Overexpression of miR-25 has been linked to tumor progression and unfavorable prognosis in breast cancer (149). miR-25 may contribute to cancer progression by targeting tumor suppressor genes and promoting cell cycle progression (150). Chen et al. found that miR-25-3p promotes proliferation by targeting tumor suppressor BTG2 (149).

Circulating miRNAs in plasma have been investigated as early detection markers for breast cancer (151). Cuk et al. identified a panel of miRNAs, including miR-155 and miR-21, whose elevated levels were associated with decreased survival rates (151). These miRNAs may serve as non-invasive biomarkers for early diagnosis and prognosis assessment (93).

While Wu et al. studied miR-9 in osteosarcoma (152), miR-9 has also been implicated in breast cancer. Elevated miR-9 levels are associated with enhanced metastasis and poor survival rates (77). miR-9 promotes epithelial-mesenchymal transition by targeting E-cadherin, facilitating tumor progression (77).

miR-21 is one of the most studied oncomiRs in breast cancer. Its overexpression is correlated with advanced tumor stage, metastasis, and reduced overall survival (153). miR-21 promotes tumor growth by targeting tumor suppressor genes such as PTEN and PDCD4 (154, 155). High miR-21 levels have been proposed as a predictor of poor prognosis (156).Similarly, miR-155 is overexpressed in breast cancer and is associated with aggressive tumor characteristics and decreased survival rates (144). Its role in promoting proliferation and inhibiting apoptosis contributes to tumor progression (73).

In contrast, miR-34a acts as a tumor suppressor and its reduced expression is linked to poor prognosis (157). Restoration of miR-34a levels inhibits tumor growth and enhances sensitivity to chemotherapy (158).

The miR-200 family is known for suppressing metastasis by inhibiting epithelial-mesenchymal transition (61). Lower expression levels of miR-200c have been associated with higher metastatic potential and worse survival outcomes (159).

miR-210 is upregulated under hypoxic conditions within tumors and is linked to poor prognosis in breast cancer patients (160). Its expression promotes angiogenesis and adaptation to hypoxia, facilitating tumor survival and progression (161).

miR-31 has been identified as a tumor suppressor, and higher levels are associated with reduced metastasis and improved survival rates (162). It inhibits multiple steps of the metastatic process, including invasion and colonization (163).

Understanding the relationship between specific miRNAs and patient survival can aid in the development of prognostic biomarkers and personalized therapies (131). miRNA expression profiling may help identify high-risk patients who could benefit from more aggressive treatment strategies (14).

miRNAs play a significant role in breast cancer progression and patient survival. Specific miRNAs such as miR-29a, miR-25, miR-21, and miR-155 are associated with poor prognosis, while others like miR-34a and miR-31 correlate with better survival outcomes. Further research into miRNA-based diagnostics and therapeutics holds promise for improving breast cancer patient management and survival rates.

### 7.2 The potential of miRNAs as prognostic biomarkers

Breast cancer prognosis remains a significant challenge due to the heterogeneity of the disease and the variability in patient outcomes (52, 164). Traditional prognostic factors, such as tumor size, lymph node status, and hormone receptor expression, do not fully capture the complexity of tumor biology (165). miRNAs, small non-coding RNAs that regulate gene expression post-transcriptionally, have emerged as promising prognostic biomarkers in breast cancer (98, 166). Their dysregulated expression in cancer tissues and presence in bodily fluids enable non-invasive prediction of disease progression and patient outcomes (14, 167).

miRNA dysregulation plays a critical role in breast cancer development and progression (12, 52). Mulrane et al. highlighted that specific miRNAs are consistently altered in breast cancer tissues, affecting cell proliferation, apoptosis, and metastasis (52). For instance, overexpression of oncomiRs such as miR-21 and miR-155 has been associated with poor prognosis and decreased overall survival (144, 153). Conversely, downregulation of tumor-suppressive miRNAs like miR-34a and miR-200c correlates with increased tumor aggressiveness and unfavorable outcomes (168, 169).

Circulating miRNAs in serum and plasma have garnered attention as minimally invasive prognostic biomarkers (90, 170). These miRNAs are remarkably stable in circulation due to their encapsulation within exosomes or association with RNA-binding proteins (89, 171). Erbes et al. demonstrated the feasibility of detecting circulating miRNAs in breast cancer patients, suggesting their potential in monitoring disease progression and response to therapy (166).

Several clinical studies have identified specific miRNAs with prognostic significance:

1. miR-21: Elevated levels of circulating miR-21 have been linked to advanced tumor stage, lymph node metastasis, and reduced survival rates (153). Its role in promoting cell proliferation and inhibiting apoptosis makes it a valuable prognostic marker.
2. miR-155: Overexpression of miR-155 is associated with aggressive breast cancer phenotypes and poor clinical outcomes (144, 172). Its prognostic value has been validated in multiple patient cohorts.
3. miR-210: As a hypoxia-induced miRNA, miR-210 levels correlate with tumor hypoxia, a condition linked to therapy resistance and poor prognosis (160, 161).
4. miR-18a and miR-200c: Low expression levels of these miRNAs have been associated with increased risk of relapse and decreased overall survival (169, 173).

Wang et al. compared the miRNA spectrum between serum and plasma, concluding that both sources are suitable for miRNA biomarker discovery (98). However, they noted that certain miRNAs may show differential stability or abundance, emphasizing the need for standardized sample processing protocols (98, 102).

While the potential of miRNAs as prognostic biomarkers is promising, several challenges need to be addressed:

1. Standardization: Variability in sample collection, RNA isolation, and detection methods can affect miRNA quantification (91, 174). Establishing standardized protocols is crucial for reproducibility.
2. Specificity and Sensitivity: Some miRNAs may not be exclusively expressed in breast cancer, necessitating the use of miRNA panels to improve specificity (93, 175).
3. Validation in Large Cohorts: Many studies have small sample sizes. Larger, multicenter studies are required to validate the prognostic value of candidate miRNAs (176).

Advancements in high-throughput technologies and bioinformatics analysis are facilitating the discovery of novel miRNA biomarkers (177, 178). Integrating miRNA profiling with other molecular data, such as gene expression and proteomics, may provide a more comprehensive prognostic model (179). Additionally, exploring the functional roles of prognostic miRNAs could uncover new therapeutic targets (**Table 1**).

**Table 1:** Breast cancer-associated miRNAs: targets and clinical relevance.

miRNAs	Expression	Targets	Clinical relevance
miR-21	Overexpression	PTEN, PDCD4, TPM1, LZTFL1	Diagnostic biomarker (serum) Poor prognosis Trastuzumab resistance
miR-155	Overexpression	SOCS1, RHOC, VHL	Diagnostic biomarker (serum/plasma) Poor prognosis Associated with triple-negative phenotype
miR-34a	Low Expression	BCL2, CDK6, Notch1	Poor prognosis Enhances chemotherapy sensitivity
miR-451	Low Expression	MDR1	Doxorubicin resistance
miR-200 family	Low Expression	ZEB1, ZEB2	Poor prognosis Associated with high metastatic potential
miR-221/222	Overexpression	p27^Kip1^, ERα	Tamoxifen resistance Poor prognosis
miR-124	Low Expression	TFAP4	Poor prognosis
miR-143	Low Expression	MAPK3	Bone metastasis marker Poor prognosis
miR-210	Overexpression	EFNA3	Hypoxia-related marker Poor prognosis HER2-positive tumor association
miR-9	Overexpression	E-cadherin	Metastasis marker Poor prognosis
miR-125b	Overexpression (circulating)	-	Chemoresistance marker Poor treatment response

## 8 Controversies in the application of mirnas

The application of miRNAs has many limitations and controversies. May Be related to the complexity of research methods, technical limitations. The problem of academic fraud in the field of miRNAs also needs attention,

The mechanisms of action of miRNAs are inherently complex and uncertain. A single miRNA can regulate hundreds of target genes, while a single gene can be co-regulated by multiple miRNAs. This complex regulatory network makes it exceedingly difficult to precisely elucidate their biological functions (180, 181). The same miRNA may play diametrically opposed roles in different tissues or disease stages, such as MIR-125B, which is downregulated as a tumor suppressor in ovarian cancer but upregulated as an oncogene in prostate cancer (180).

There are also limitations to the use of mirnas as biomarkers. Although the stability of miRNAs in bodily fluids gives them potential as disease markers, the issue of detection standardization remains unresolved. Different studies employ varying sample processing methods and extraction techniques, making results unconvincing (91). For example, miR-375 has been associated with pancreatic β-cell damage. However, studies have found that only about 1% of its plasma concentration originates from β-cells, its sensitivity and specificity as a circulating biomarker for β-cell damage are insufficient, and its clinical application is limited (181).

In the field of miRNA research, the issue of academic fraud cannot be ignored. Some studies have engaged in data tampering and fabricated results. Such fraud not only distorts the true value of miRNAs as diagnostic and therapeutic targets for breast cancer, but also wastes research resources and misleads the subsequent research direction. Such behaviors seriously undermine the credibility of miRNA research in breast cancer precision medicine and hinder the process from basic research to clinical translation. Therefore, establishing a strict data verification mechanism, promoting the standardization of experimental methods, and strengthening academic supervision are the key to curbing academic fraud in this field and ensuring the authenticity of research.

## 9 Discussion

Breast cancer is one of the most common malignancies among women worldwide, remains one of the leading causes of cancer-related mortality (1, 3, 78). The pathogenesis of breast cancer is complex, involving genetic, endocrine, environmental, and other factors, and its high heterogeneity poses significant challenges for diagnosis, treatment, and prognosis (5, 6, 182, 183). Therefore, there is an urgent need to identify new biomarkers and therapeutic targets to improve early diagnosis rates and treatment outcomes for breast cancer.

miRNAs have garnered significant attention for their dual roles in cancer which acting as both tumor suppressors and oncogenes (11). In breast cancer, an increasing number of studies have shown that dysregulation of specific miRNAs is closely related to the initiation, progression, invasion, and metastasis of breast cancer (12, 184). Given the significant role of miRNAs in breast cancer, this article aims to systematically review the latest research progress on the role of miRNAs in the diagnosis and treatment of breast cancer.

miRNAs exhibit complex regulatory functions in breast cancer cell proliferation, apoptosis, invasion and metastasis (12, 28, 184, 185). miR-10b targets E-cadherin, a key cell-cell adhesion molecule, leading to decreased cell adhesion and increased cell motility, facilitating metastatic spread (65). Conversely, the miR-200 family functions as metastasis suppressors by targeting transcriptional repressors ZEB1 and ZEB2, which are key regulators of EMT (74).

Traditional diagnostic methods have different limitations. Circulating miRNAs in serum and plasma have been extensively studied as potential diagnostic tools for breast cancer due to their stability in bodily fluids and their altered expression profiles in cancer patients. miR-1246 and miR-1307-3p, which could effectively detect breast cancer at early stages (81). In addition, several studies have identified miRNAs differentially expressed in breast cancer patients, such as miR-21, miR-221, miR-195 and so on (26, 87). There are broad prospects for miRNA to be a diagnostic biomarker of breast cancer. Specific miRNAs are associated with survival in patients with breast cancer and may serve as prognostic biomarkers. Specifically, miRNAs such as miR-21, miR-155, and miR-34a have been shown to correlate with patient outcomes, highlighting their potential in guiding therapeutic strategies.

Multiple therapies, such as miRNA replacement therapy, are being developed to improve the survival of breast cancer patients (113). Several clinical trials to assess the safety and efficacy of miRNA therapy are ongoing. However, miRNA therapy faces challenges such as immune activation, off-target effects, and delivery efficiency (127). Our analyses revealed that miRNAs can also interact with conventional therapies such as radiotherapy and chemotherapy by influencing treatment response and resistance mechanisms. Such as the overexpression of miR-451 in MCF-7 breast cancer cells led to increased sensitivity to doxorubicin by targeting the multidrug resistance protein MDR1 (133).

Due to sample handling, patient heterogeneity, and detection methods, the reproducibility of miRNAs-related studies may be low (91). Therefore, the credibility and value of the research are questioned and the clinical transformation is faced with challenges (186). Future research should address these issues. With continued research and refinement of analytical methods, miRNAs may soon become integral to personalized breast cancer treatment strategies, improving patient outcomes through more targeted and effective management.

## 10 Conclusion

In summary, our study systematically reviews the latest research progress on the role of miRNAs in the diagnosis and treatment of breast cancer, with a focus on their application as tumor markers in early diagnosis, molecular subtyping, therapeutic response monitoring, and the prognostic evaluation, and constructs a complete cognitive framework. Simultaneously, we explored the perspective of clinical application, in-depth analysis of the problems faced when translating its detection technology and targeted therapy into routine clinical applications, such as the standardization of detection methods, the safety and effectiveness of treatment, etc. it provides a more targeted guidance for clinical practice.
